# Multi-Layered Composites of Natural Rubber (NR) and Bismuth Oxide (Bi_2_O_3_) with Enhanced X-ray Shielding and Mechanical Properties

**DOI:** 10.3390/polym15122717

**Published:** 2023-06-17

**Authors:** Donruedee Toyen, Ekachai Wimolmala, Kiadtisak Saenboonruang

**Affiliations:** 1 Department of Materials Science, Faculty of Science, Kasetsart University, Bangkok 10900, Thailand; donruedee.toyen@ku.th; 2 Special Research Unit of Radiation Technology for Advanced Materials (RTAM), Faculty of Science, Kasetsart University, Bangkok 10900, Thailand; 3 Polymer PROcessing and Flow (P-PROF) Research Group, Division of Materials Technology, School of Energy, Environment and Materials, King Mongkut’s University of Technology Thonburi, Bangkok 10140, Thailand; ekachai.wim@kmutt.ac.th; 4 Department of Applied Radiation and Isotopes, Faculty of Science, Kasetsart University, Bangkok 10900, Thailand; 5 Specialized Center of Rubber and Polymer Materials in Agriculture and Industry (RPM), Faculty of Science, Kasetsart University, Bangkok 10900, Thailand

**Keywords:** natural rubber, bismuth oxide, multi-layered structure, mechanical properties, X-ray shielding, thermal aging, composites

## Abstract

Due to rapid increases in the utilization of radiation and nuclear technologies, effective and suitable radiation-shielding materials have become one of the most sought-after options to protect users and the public from excessive exposure to the radiation. However, most radiation-shielding materials have greatly reduced mechanical properties after the addition of fillers, resulting in their limited useability and shortened lifetime. Therefore, this work aimed to alleviate such drawbacks/limitations by exploring a possible method to simultaneously enhance both the X-ray shielding and mechanical properties of bismuth oxide (Bi_2_O_3_)/natural rubber (NR) composites through multi-layered structures, with varying (1–5) layers and a total combined thickness of 10 mm. To correctly determine the effects of the multi-layered structures on the properties of NR composites, the formulation and layer configuration for all multi-layered samples were tailored such that their theoretical X-ray shielding properties were equal to those of a single-layered sample that contained 200 phr Bi_2_O_3_. The results indicated that the multi-layered Bi_2_O_3_/NR composites with neat NR sheets on both outer layers (sample-D, sample-F, sample-H, and sample-I) had noticeably higher tensile strength and elongation at break than those of the other designs. Furthermore, all multi-layered samples (sample-B to sample-I), regardless of the layer structure, had enhanced X-ray shielding properties compared to those with a single layer (sample-A), as shown by their higher values of the linear attenuation coefficient (µ) and lead equivalence (Pb_eq_) and the lower value of the half-value layer (HVL) in the former. This work also determined the effects of thermal aging on relevant properties for all samples, with the results revealing that all the thermal-aged composites had higher values for the tensile modulus but lower values for the swelling percentage, tensile strength, and elongation at break, compared with the non-aged composites. Hence, based on the overall outcomes from this work, it could be concluded that the worrisome decreases in mechanical properties of the common single-layered NR composites after the addition of Bi_2_O_3_ could be prevented/reduced by introducing appropriate multi-layered structures, which would not only widen potential applications but also prolong the lifetime of the composites.

## 1. Introduction

Radiation knowledge and technologies, especially those involving X-rays, have been increasingly utilized in various applications, including X-ray imaging [1], elemental analysis for archaeological objects and agricultural products [2,3], and the identification of counterfeit medicines and artworks [4,5]. However, excessive exposure to X-rays could fatally harm users and the public, with the symptoms varying from mild nausea, headache, and diarrhea to dysfunctional organs, cancers, permanent injuries, and even death [6]. In order to protect and/or reduce risks of users from excessive exposure during the operations and services, stringent safety protocols through the management of working time and distance, as well as the utilization of appropriate shielding equipment, must be strictly followed in all radiation-related facilities [7].

In particular for shielding equipment, which primarily aims to attenuate the intensity of incoming radiation to within safe limits, selection of the main materials depends on several factors, including the type and energy level of the relevant radiation, the nature and requirement of the intended usage, and the budget to acquire the equipment. For example, applications requiring high strength and rigidity could rely on polyethylene (PE) [8], polyvinyl chloride (PVC) [9,10], glass [11], and concrete [12] as main materials, while natural rubber (NR) [13,14], silicone rubber (SR) [15], and styrene-butadiene rubber (SBR) [16] would be preferrable choices for applications that require high flexibility and comfort. Nonetheless, these mentioned materials are not readily suitable for use as X-ray shielding materials in their pristine forms due to their compositional lack of heavy-metal compounds, such as lead (Pb) and lead oxide (Pb_3_O_4_). Pb compounds are useful in such applications because they have relatively higher interaction probabilities with incoming X-rays through two main mechanisms (photoelectric absorption and Compton scattering), resulting in larger energy transfers and more efficient X-ray attenuation than those achieved using lighter elements [17]. Examples of some materials relying on Pb compounds as X-ray protective fillers are Pb_3_O_4_/epoxy [18] and Pb/concrete [19].

Despite the effectiveness and common use of Pb compounds as protective fillers for X-rays, the high toxicity of Pb compounds has prevented/limited their useability and processibility due to growing concerns related to health and eco-systems [20]. To cope with such challenging demands, other heavy-metal compounds, such as bismuth oxide (Bi_2_O_3_), tungsten oxide (WO_3_), barium sulfate (BaSO_4_), and gadolinium oxide (Gd_2_O_3_), which are relatively safer than Pb compounds, have drawn great attention from product developers to explore their potential as alternative protective fillers for X-rays [21,22,23,24]. For example, Thumwong et al. revealed that the addition of 95–140 parts per hundred parts of rubber by weight (phr) nano-Bi_2_O_3_ or 105–120 phr nano-BaSO_4_ in 0.25-mm natural rubber latex (NRL) gloves was sufficient to attenuate 60 kV and 100 kV X-rays with equal efficiency to a 0.02-mm Pb sheet (a common specification for commercial X-ray shielding latex gloves) [25]. Furthermore, Poltabtim et al. showed that the addition of 25 phr Gd_2_O_3_ in self-healing NR composites lowered the half value layer (HVL) from 1.12 cm and 2.50 cm in a pristine NR to just 0.36 cm and 0.65 cm in Gd_2_O_3_/NR composites for the attenuation of 60 kV and 100 kV X-rays, respectively, implying enhanced X-ray shielding abilities after the addition of Gd_2_O_3_ [26].

While it is generally the case that the ability of composites to attenuate X-rays improves with increasing contents of protective fillers [21,22,23,24,25,26], unpleasant side-effects may occur, such as particle agglomerations, voids, cracks, and phase separations in the matrix, leading to substantially lower mechanical and physical properties of the composites, often to levels that the materials become unsuitable for use in actual applications. For example, the values of tensile strength and elongation at break of NRL gloves were reduced from 22 MPa and 1250%, respectively, in pristine NRL gloves to just 5 MPa and 700%, respectively, in 200 phr Bi_2_O_3_/NRL gloves, with these latter values being lower than the mechanical requirements for non-aged medical examination latex gloves (ASTM D3578-19) [25,27]. Another report revealed that the same mechanical parameters for eucalyptus pulp/NR composites were reduced from mean values (±standard deviation) of 10.02 ± 0.88 MPa and 733 ± 5%, respectively, in those without BaSO_4_ to 8.39 ± 1.07 MPa and 558 ± 19%, respectively, in those containing 22.46 wt% BaSO_4_, confirming the negative effects of such fillers on the mechanical properties of the composites [28]. Hence, clearly, a novel and improved procedure/method is required to limit reductions in the mechanical properties of the shielding composites after the addition of protective fillers.

Several attempts have been made to address the above concerns. For example, the NR composites could be reinforced by combining the composites with either natural or synthetic fibers, such as sisal, oil palm, pineapple leaves, and glass fibers [29,30,31]. However, this method usually requires additional procedures to optimize manufacturing processes in order to accommodate the added fibers, which could lengthen the times and costs associated with sample preparation. In addition to fibers, chemically treated fillers using silane coupling agents are one of the common and effective methods used to improve the surface compatibility, particle distribution, and hence the mechanical properties of the composites [32]. However, this chemical method has raised serious concerns by scientific communities and activists as silane coupling agents are highly flammable and toxic, limiting their use due to the current demands for greener technologies [33,34]. Another interesting method (but with very limited available data) is to explore the advantages of multi-layered products. For this method, layers of neat NR and NR composites containing X-ray protective fillers (Bi_2_O_3_ in the current work) are placed consecutively on top of each other, with the total numbers of layers and structures being designed to meet specific requirements. With such a design, the neat NR layers, which generally have improved abilities to transfer and withstand external forces than those of NR composites, could act as main force absorbers, subsequently improving the overall mechanical properties of the composites [35]. Some examples of multi-layered composites reported include electromagnetic interference (EMI) shielding materials based on iron/crumb rubber composites and SR/MXene/Fe_3_O_4_ composites, for which the results showed simultaneous enhancements in EMI shielding effectiveness and the overall tensile properties of the composites with the multi-layered structures [36,37]. Additionally, a theoretical study reported the potential enhancement in X-ray shielding capabilities of multi-layered Bi_2_O_3_/NR composites (determined using a web-based simulation software, namely XCOM) compared with those of a single-layered one, with the outcomes of experimental investigations required to confirm their validity [38].

As mentioned above, the reduced mechanical properties, as well as the limited data availability of X-ray shielding NR composites with multi-layered structures, have driven the need for wider and more thorough investigations. Hence, the current work comprehensively explored the potential advantages of multi-layered structures in X-ray shielding Bi_2_O_3_/NR composites by comparing relevant properties, consisting of density, morphology, tensile property, and X-ray attenuation, of single-layered NR composites (sample-A) to those of multi-layered NR composites (sample-B to sample-I). In addition, the numbers (maximum of 5 layers and a total combined thickness of 10 mm) and arrangements of neat NR and Bi_2_O_3_/NR layers were varied to determine behavioral trends and suitable structures for actual use. Notably, the formulations for each layer of the multi-layered NR composites were determined such that their final X-ray shielding properties were theoretically the same as those of a single-layered sample containing 200 phr Bi_2_O_3_. In addition, this work determined the effects of thermal aging by comparing the swelling and mechanical properties of thermal-aged samples to those of non-aged ones [39]. The outcomes from this work not only present innovative and greener methods to improve mechanical and X-ray shielding properties of Bi_2_O_3_/NR composites through multi-layered structures but also provide knowledge and techniques as the bases for the development of other similar materials.

## 2. Experimental

### 2.1. Materials and Chemicals

The names, contents, and roles of the chemicals used during sample preparation are shown in Table 1. Bi_2_O_3_ particles, with a mean (±standard deviation) particle size of 27.4 ± 8.2 µm, were purchased from Shanghai Ruizheng Chemical Technology Co., Ltd. (Shanghai, China) and used without any further modification [9]. Natural rubber (STR20) and other chemicals were supplied by the Rubber Authority of Thailand (RAOT; Bangkok, Thailand). Notably, the formulations and procedures used in this work were based on our previous study involving the development of metal oxide/NR composites for gamma-ray shielding [40].

### 2.2. Determination of Bi_2_O_3_ Contents in Each Layer of Multi-Layered Bi_2_O_3_/NR Composites

**Figure 1:**
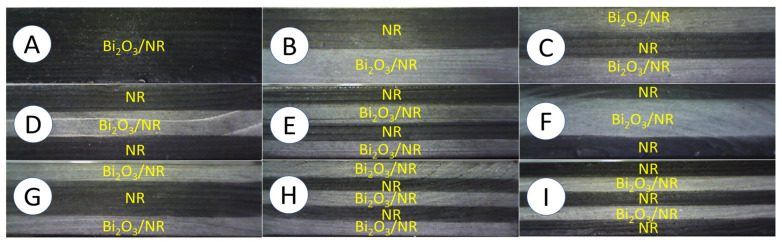
Optical images showing cross sections of 9 distinct structures of 10-mm Bi_2_O_3_/NR composites, where letters (**A** to **I**) enclosed in circles are sample codes listed in Table 2.

Table 2 and Figure 1 show the details and schemes, respectively, of the 9 distinct structures for 10-mm Bi_2_O_3_/NR composites with varying numbers (1–5) of layers and varying Bi_2_O_3_ contents for each layer. The Bi_2_O_3_ contents for each Bi_2_O_3_/NR layer and design were determined with a preliminary requirement that the final X-ray shielding properties of all multi-layered samples must be theoretically equal to those of a single-layered sample containing 200 phr Bi_2_O_3_ (sample-A). This constraint was used to correctly determine the effects of the multi-layered structures on the actual mechanical and X-ray shielding properties of the Bi_2_O_3_/NR composites. To meet the above requirement, the number and density of Bi_2_O_3_ particles, which directly related to the bulk densities (ρ_sample_), of all samples was kept the same, regardless of the layer structure, with the theoretical determination of the bulk density and Bi_2_O_3_ content determined using Equation (1) [13]:

(1) $$\rho_{sample}=\frac{1}{N}\sum_{i=1}^{N}\left[\left(C_{NR}+C_{Bi,i}\right)/\left(\left(\frac{C_{NR}}{\rho_{NR}}\right)+\left(\frac{C_{Bi,i}}{\rho_{Bi}}\right)\right)\right].$$

where ρ_NR_ is the density of neat NR (0.92 g/cm^3^) [38], ρ_Bi_ is the density of Bi_2_O_3_ (8.90 g/cm^3^) [38], N is the number of layers (1–5), C_NR_ is the content of NR (fixed at 100 phr for all layers), and C_Bi,i_ is the content of Bi_2_O_3_ in the i^th^ layer (phr). According to Equation (1), sample-A would have a theoretical bulk density of 2.28 g/cm^3^, which was then used as a reference value for the determination of C_Bi,i_ in sample-B to sample-I. Notably, the Bi_2_O_3_ content for sample-A was limited to 200 phr in this work as this content would imply a value of C_Bi,2_ in sample-D to be as high as 1034 phr, which would already lead to extreme difficulties during the compounding and curing processes of the respective layer.

### 2.3. Preparation of Bi_2_O_3_/NR Compounds

The NR was first masticated using a rubber kneader for 4 min and then compounded with ZnO and stearic acid for 2 min. After thorough mixing, paraffinic wax, sulfur, MBT, and DPG were orderly added to the compound and the mixing continued for a further 3–4 min. Next, the prepared NR compounds were rolled into sheets with specific thicknesses (as outlined in Table 2) using a two-roll mill (Yong Fong Machinery Co., Ltd.; Samutsakorn, Thailand). Later, the sheets were cut into square shapes with dimensions of 16 cm × 16 cm and kept in a refrigerator before further processing.

### 2.4. Cure Characteristics

The scorch times (t_s2_), cure times (t_c10_, t_c50_, t_c90_, and t_c95_), and torque characteristics (M_L_, M_H_, and M_H_ − M_L_) for each Bi_2_O_3_/NR layer were evaluated using a moving die rheometer (TECH PRO; Columbia city, IN, USA) at 150 °C, following the ASTM D2084 [41,42]. Notably, the shortest t_c90_—the time the NR specimen needed to achieve 90% of its respective maximum torque (M_H_)—among all NR layers was selected as the time to cure every multi-layered sample. The rationale for using the shortest t_c90_ was that longer cure times would make some layers, especially neat NR, too rigid from over-curing, leading to a lower adhesive ability between layers and the subsequent deterioration of the potential role of the neat NR to transfer external forces from the Bi_2_O_3_/NR layers.

### 2.5. Preparation of Multi-Layered Bi_2_O_3_/NR Composites

To prepare the multi-layered Bi_2_O_3_/NR composites, the prepared neat NR and Bi_2_O_3_/NR layers obtained from Section 2.3 were stacked according to the schemes shown in Figure 1, with a total combined thickness of 10 mm. Then, the stacked NR layers were vulcanized and pressed using a hydraulic press at 150 °C and a pressure of 120 psi, with the cure time obtained from Section 2.4 (t_c90_ of neat NR).

### 2.6. Thermal Aging on Multi-Layered Bi_2_O_3_/NR Composites

For the determination of thermal aging effects on the swelling and mechanical properties, the multi-layered Bi_2_O_3_/NR composites were placed in a hot-air oven (ED/FD Binder; Scientific Product Co., Ltd.; Bangkok, Thailand) at 70 °C for 168 h, following the ASTM D573-04 [43] standard testing procedure.

### 2.7. Characterization of Multi-Layered Bi_2_O_3_/NR Composites

#### 2.7.1. Density, Morphology, Surface Roughness, and Swelling Tests

The densities (ρ) of all multi-layered Bi_2_O_3_/NR composites were determined by finding the fraction of mass (M), determined using a 4-digit-accuracy scale (Practum224-1S; Sartorius; Göttingen, Germany), over volume (V), determined using a set of digital vernier calipers with 0.02-mm precision (Duratool; Bangkok, Thailand). The morphologies were determined using a scanning electron microscope with energy dispersive X-rays (SEM-EDX) (SU3500; Hitachi; Tokyo, Japan). All samples were coated with a layer of gold (0.2 mm thick) using a sputter coater (Mini Sputter Coater/Glow Discharge System; SC7620; Quorum, Laughton, UK) prior to SEM images being taken. The roughness of fractured surface for each sample was also characterized using a surface-roughness tester (SURFTEST SV-3100; Mitutoyo; Kawasaki, Japan) with a testing speed of 100 µm/s, for which the samples were snapped after being submerged in liquid nitrogen, prior to the testing.

In addition, to evaluate the ability of the samples to resist the absorption of solvent into the matrix, as well as the degree of crosslinking, the values of the swelling percentage (swelling %) of the multi-layered Bi_2_O_3_/NR composites were determined by immersing 2 cm circular samples in toluene for 24 h in a dark chamber. Then, the weights of the samples, both prior (w_1_) and after (w_2_) the immersion, were measured using a 4-digit-accuracy scale and the values of swelling % were determined, based on Equation (2):

(2) $$Swelling\;\left(\%\right)=\frac{w_{2}-w_{1}}{w_{1}}\times 100\%$$

Notably, the density, surface roughness, and swelling measurements were conducted with at least 3 repetitions.

#### 2.7.2. X-Ray Shielding Properties

The X-ray shielding properties of the multi-layered Bi_2_O_3_/NR composites were evaluated at the Secondary Standard Dosimetry Laboratory (SSDL), the Office of Atoms for Peace (OAP) (Bangkok, Thailand). The relevant shielding parameters of interest were the X-ray transmission fraction (I/I_0_), the linear attenuation coefficient (µ), the half-value layer (HVL), and the lead equivalence (Pb_eq_), which were determined using Equations (3)–(5) [25]:

(3) $$\mu =-\frac{ln(I/I_{0})}{x}$$

(4) $$HVL=\frac{ln(2)}{\mu }$$

(5) $${Pb}_{eq}=\frac{\mu \cdot x}{\mu_{Pb}}$$

where x and µ_Pb_ are the thickness of the sample and the linear attenuation coefficient of a pure Pb sheet, respectively. Since the value of µ depends strongly on the X-ray energy, the µ_Pb_ used for the calculation of Pb_eq_ was experimentally determined using a 0.1-mm Pb sheet and the same setup as for the Bi_2_O_3_/NR composites. Then, the obtained I/I_0_ values from the irradiation of 60 kV, 100 kV, and 150 kV X-rays were used to determine the respective µ_Pb_ values, which were 67.04 cm^−1^, 25.75 cm^−1^, and 31.12 cm^−1^, respectively.

Additional details on the setup of the X-ray shielding measurement have been provided elsewhere [25]. In summary, 1-mm collimated X-rays were emitted from an X-ray tube with varying supplied voltages (60 kV, 100 kV, and 150 kV) that corresponded to average X-ray energies of 47.9 keV, 83.3 keV, and 118.0 keV, respectively. The X-ray beam was pointed directly at the center of the multi-layered Bi_2_O_3_/NR samples, which were placed at the midpoint between the X-ray tube and the detector that were 100 cm apart. The initial (I_0_) as well as the transmitted (I) X-ray intensities were detected and counted using a free air ionization chamber (Korea Research Institute of Standards and Science, KRISS; Daejeon, Republic of Korea). Notably, the X-ray shielding measurements were conducted with at least 5 repetitions for each sample.

#### 2.7.3. Mechanical Properties

The mechanical properties, consisting of the tensile modulus at 100% elongation (M100), tensile strength (TS), and elongation at break (EB), of all multi-layered Bi_2_O_3_/NR composites were determined using a Universal Testing Machine (TM-G5K; TM Tech Testing Co., Ltd.; Bangkok, Thailand), following the ASTM D412-16 standard testing procedure [44], with at least 3 repetitions per sample. The tensile testing speed used in this test was 500 mm/min.

## 3. Results and Discussion

### 3.1. Cure and Torque Characteristics

The torque characteristics, consisting of minimum torque (M_L_), maximum torque (M_H_), and torque difference (M_H_ − M_L_), for neat NR and Bi_2_O_3_/NR composites with varying Bi_2_O_3_ contents from 200 to 1034 phr are shown in Table 3. The results indicated that M_L_ and M_H_, which have strong correlations with the viscosity and rigidity of the composites [41], increased with increasing Bi_2_O_3_ contents. These results were due to the high rigidity of the added Bi_2_O_3_ particles that subsequently increased the overall rigidity of the composites. In addition, the added Bi_2_O_3_ particles restricted the molecular motion and flow of the NR, leading to increased viscosity of the NR composites containing Bi_2_O_3_ [45]. Table 3 shows that the values of M_H_ − M_L_, which are positively related to the crosslink density of the composites, increased with increasing Bi_2_O_3_ contents [46]. The increases in M_H_ − M_L_ were mainly due to Bi_2_O_3_ particles acting as a co-activator during the chemical vulcanization process that resulted in better bonding between the NR molecular chains and sulfur, resulting in an increased crosslink density and thus the observed M_H_ − M_L_ value [40,47]. These findings agreed well with the previous report of metal oxide/NR composites in gamma shielding that also showed increases in M_H_ − M_L_ values after the addition of metal oxides (Bi_2_O_3_, WO_3_, and Fe_3_O_4_) [40].

The cure characteristics of the neat NR and Bi_2_O_3_/NR composites, consisting of the scorch time (t_s2_) and cure times (t_c10_, t_c50_, t_c90_, and t_c95_), are shown in Table 4. The results indicated that t_s2_ values were roughly the same up to the initial addition of 200 phr Bi_2_O_3_, but then decreased at higher contents. The decreases in t_s2_ could have been due to the added Bi_2_O_3_ particles generating frictional heat in the NR compounds from the shearing motions between the Bi_2_O_3_ and NR molecular chains during the MDR testing process that led to a more rapid initiation of the curing process and subsequently lower t_s2_ values [41,48,49]. On the other hand, Table 4 shows that all cure times (t_c10_–t_c95_) increased with increasing Bi_2_O_3_ contents, which could have been due to the added Bi_2_O_3_ particles restricting/preventing the action of the main and more effective accelerators and crosslinker (sulfur), resulting in prolonged cure times.

**Figure 2:**
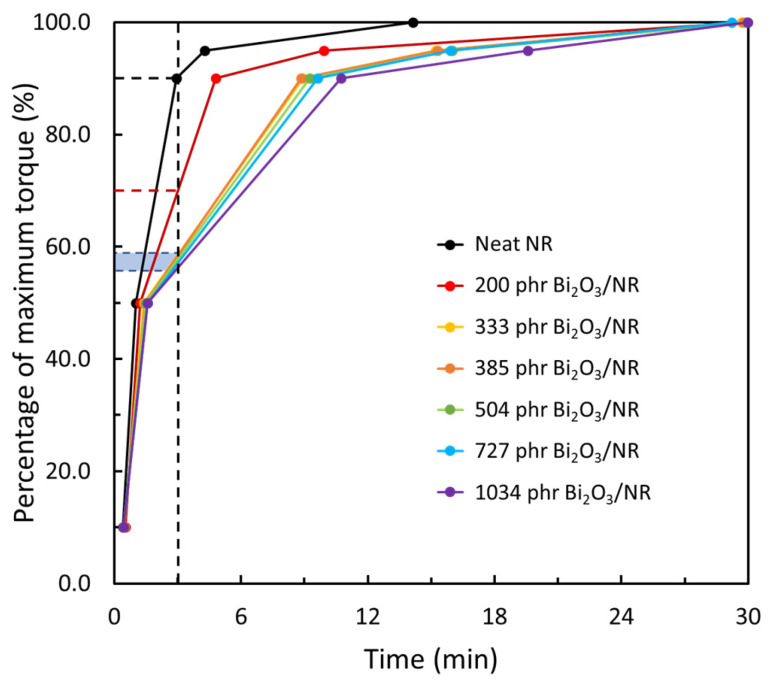
Correlations between cure times and percentages of maximum torque (%M_H_) for neat NR and Bi_2_O_3_/NR composites with varying Bi_2_O_3_ contents from 200 to 1034 phr.

As explained in Section 2.4, the shortest t_c90_ value shown in Table 4 (2.94 min for a neat NR layer) was used as the cure time to vulcanize and prepare all the multi-layered samples. As a result, NR layers containing Bi_2_O_3_ particles would have torques/crosslink densities less than 90% of their respective maximum values. To estimate the degree of crosslink density for each layer, correlations between the cure time and percentage of M_H_ for each composite are shown in Figure 2. The results indicated that, while a neat NR layer reached 90% of its M_H_ (~0.66 N·m), other Bi_2_O_3_/NR layers containing 200 phr and 333–1034 phr Bi_2_O_3_ could achieve only ~70% and ~56–59%, respectively, of their M_H_ values, leaving room for further curing with the presence of additional heat.

### 3.2. Density, Morphology, and Surface Roughness

**Figure 3:**
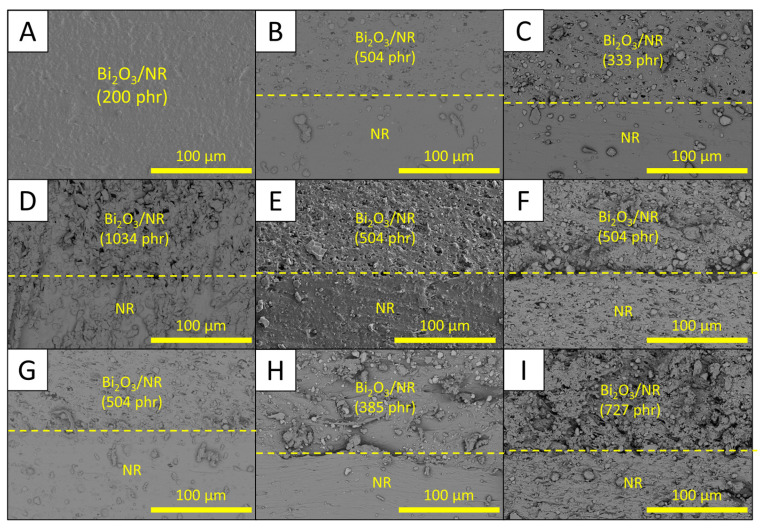
Micrograph images showing morphologies of single-layered (sample-A) and multi-layered (sample-B to sample-I) Bi_2_O_3_/NR composites. Letters enclosed in squares are sample codes listed in Table 2 and the images were taken at adjacent layers (2 layers) of neat NR and Bi_2_O_3_/NR layers for each sample.

The densities of all single-layered and multi-layered Bi_2_O_3_/NR composites are shown in Table 5, which indicates that the densities of the samples were in the range of 1.96–2.04 g/cm^3^, which were less than the calculated theoretical value of 2.28 g/cm^3^ (Equation (1)). These small deviations in the measured densities, as well as being lower than the theoretical value, could have been because of several factors, such as the expansion in dimensions of the samples due to the heat during the curing process, as well as the creations of voids and phase separation due to poor interfacial compatibility between the NR matrix and Bi_2_O_3_ (Figure 3). In addition, Figure 3 shows the morphologies of cross section for all samples (without fracture), revealing that the layers of the neat NR and Bi_2_O_3_/NR composites were well-connected to each other, without any noticeable empty spaces or cracks between the layers. However, the layers containing high Bi_2_O_3_ contents (sample-B to sample-I) clearly showed more particle agglomerations than sample-A, which could have been due to the increased filler–filler interactions from having higher Bi_2_O_3_ contents that prevented the dispersion of Bi_2_O_3_ in the NR matrix [21].

In addition, the roughness of fractured surfaces for each design was also determined. The results as shown in Table 6 indicated that the roughness of neat NR layers for all samples were in the range of 1.2–17.5 µm, with the average value being 6.3 µm, while the roughness of Bi_2_O_3_/NR layers were in the range of 4.6–29.2 µm, with the average value being 17.8 µm. The results clearly showed that the surface roughness of neat NR layers was relatively lower than those of layers containing Bi_2_O_3_. These behaviors were observed due to the presence/disappearance of Bi_2_O_3_ particles at the fractured surfaces of Bi_2_O_3_/NR layers after the snap, resulting in more profile height deviations from the mean line in comparison to those of neat NR layers [50]. In addition, the higher surface roughness of fractured surfaces implied the poor interfacial compatibility between NR matrix and Bi_2_O_3_ particles, which agreed with morphological properties of the samples shown in Figure 3. It should be noted that the surface roughness of sample-I for neat NR and Bi_2_O_3_/NR layers were relatively smaller and closer to each other than those observed in sample-B to sample-H, which could be due to the thinnest layers of sample-I (2 mm) as well as the much higher rigidity of Bi_2_O_3_/NR layers than that of neat NR that resulted in Bi_2_O_3_ particles and Bi_2_O_3_/NR compounds potentially penetrating into neat NR layers during the sample preparation.

### 3.3. Swelling Behavior

**Figure 4:**
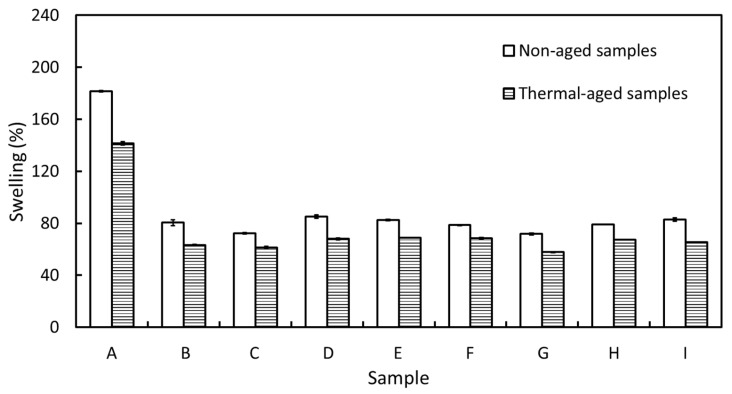
Percentages of swelling for all multi-layered Bi_2_O_3_/NR composites after immersion in toluene for 24 h. The error bars represent standard deviations of the mean and the letters in *x*-axis (A to I) are sample codes listed in Table 2.

The percentages of swelling (%swelling), after immersion in toluene, for all multi-layered Bi_2_O_3_/NR composites are shown in Figure 4, indicating that the single-layered Bi_2_O_3_/NR composites (sample-A) had a higher %swelling (182%) than those of the multi-layered samples (sample-B–sample-I) that had values in the range of 75–80%. The lower %swelling in the latter could have been due to their larger crosslink densities, as well as the dilution effects of the added Bi_2_O_3_ particles that led to reduced ability of the NR matrix to absorb toluene [51]. Additionally, the effects of thermal aging on the swelling properties of all samples were investigated and the results are shown in Figure 4, indicating that the %swelling values for all the thermal-aged samples were lower than those of the non-aged ones. This could have been due to the NR composites continuing their curing process from additional heat that enabled the remaining chemicals to continue vulcanization, resulting in increased crosslink densities and subsequently lower %swelling [52]. Notably, since all the Bi_2_O_3_/NR layers had varying degrees of crosslink density that were less than 90% of their respective maximum values (Figure 2), all the samples had substantial increases in their crosslink densities after thermal aging, thus lowering their respective %swelling values.

### 3.4. X-Ray Shielding Properties

**Figure 5:**
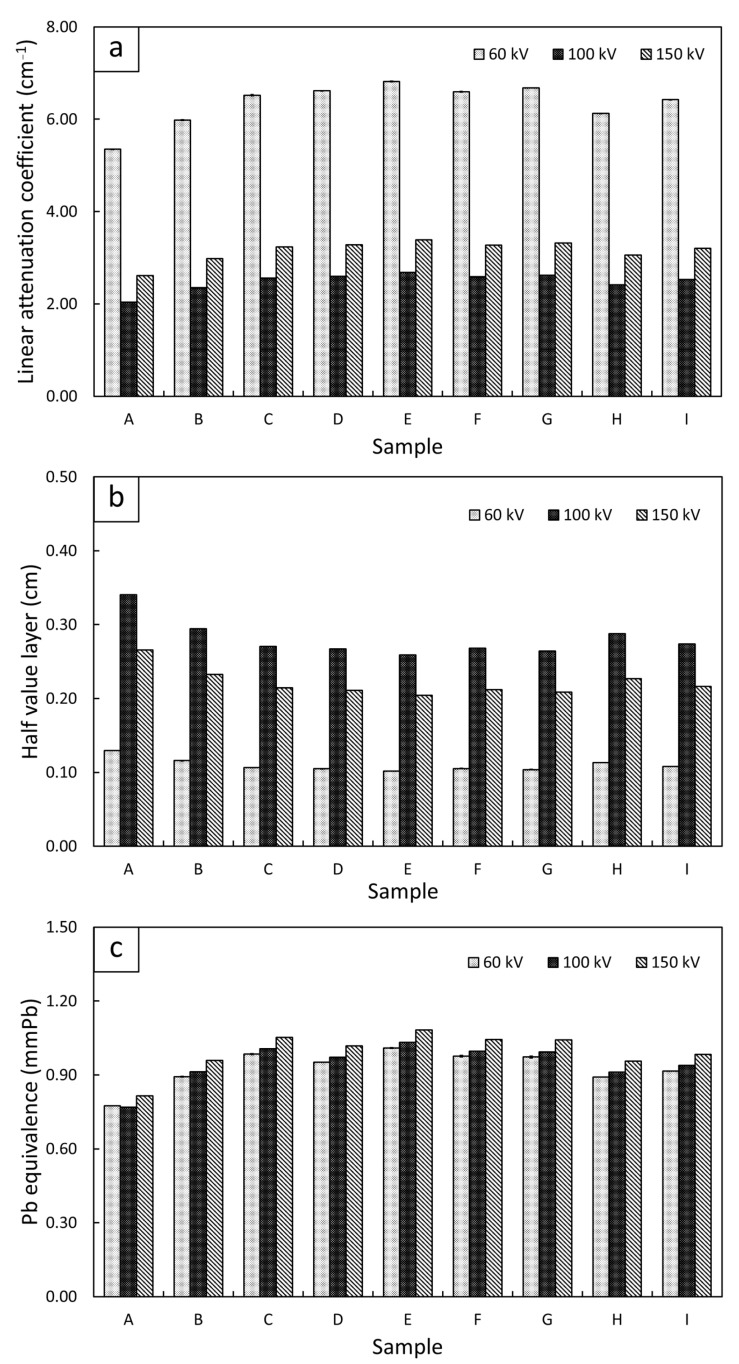
X-ray shielding properties of single-layered (sample-A) and multi-layered (sample-B to sample-I) Bi_2_O_3_/NR composites: (**a**) linear attenuation coefficient (µ), (**b**) half value layer (HVL), and (**c**) Pb equivalence (Pb_eq_). The high voltage supplied to the X-ray tube during measurement varied from 60 to 100 and 150 kV, which provided average X-ray energies of 47.9, 83.3, and 118.0 keV, respectively. It should be noted that the standard deviations of the mean for all plots were much smaller than their respective mean values, resulting in negligible error bars in the plots.

Figure 5 shows the X-ray shielding properties, consisting of the linear attenuation coefficient (µ), the half value layer (HVL), and the Pb equivalence (Pb_eq_), of the single-layered (sample-A) and multi-layered (sample-B to sample-I) Bi_2_O_3_/NR composites, determined at the supplied voltages of 60 kV, 100 kV, and 150 kV. The results indicated that all multi-layered samples had higher overall X-ray shielding properties than those of the single-layered sample, which was confirmed by their higher values of µ and Pb_eq_ and their lower values of HVL. The X-ray shielding enhancement in the multi-layered samples could have been because the multi-layered samples had thinner Bi_2_O_3_/NR layers (2.0–5.0 mm) than the single-layered one (10.0 mm), resulting in lower values of build-up factors (B) and subsequently transmitted X-rays (I), as shown in Equation (6). Notably, the build-up factor (B), defined as the fraction of the total dose over the un-scattered dose, depends on two main factors, namely the X-ray energy (E) and the material thickness (x), as shown in Equation (7) [53,54]:

(6) $$B(E,x)=\frac{I}{I_{0}}e^{\mu x}$$

(7) $$B\left(E,x\right)=1+B_{1}µxe^{B_{2}\mu x}$$

where B_1_ and B_2_ are the energy-dependent fitting parameters. As depicted in Equation (7), the thicker layer of the single-layered Bi_2_O_3_/NR composites in sample-A would have a higher B value that resulted in a larger number of transmitted X-rays (I) and subsequently of the measured µ.

**Figure 6:**
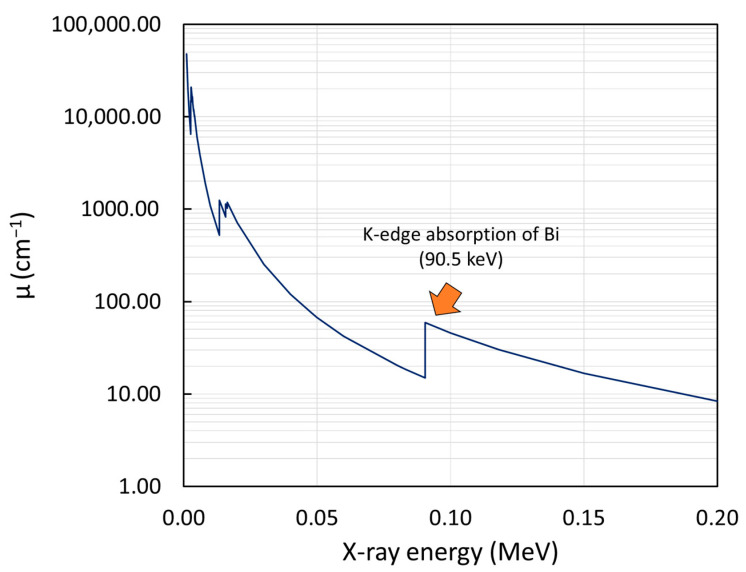
Linear attenuation coefficient (µ) of Bi_2_O_3_ at the X-ray energies in the range 0.001–0.20 MeV, generated using XCOM [55].

Another interesting result from Figure 5 was that all the samples attenuated the 60 kV X-rays with the highest levels of efficiency among all the X-ray energies, due to the higher interaction probabilities between the samples and incoming 60 kV X-rays. This finding was supported by the results shown in Figure 6, which was generated using XCOM [55], indicating that the µ value of Bi_2_O_3_ at the 47.9 keV (60 kV) X-rays was higher than those at the 83.3 keV (100 kV) and 118 keV (150 kV) X-rays, resulting in a higher interaction probability and thus a greater attenuation ability of the materials with the 60 kV X-rays. However, Figure 6 indicates a sudden increase in µ for the 90.5 keV X-rays. This uncharacteristically high value of µ at this energy was due to the energy of the incoming X-rays being just above the biding energy of electrons in the K-shell of the Bi atoms, resulting in substantially enhanced probabilities of X-ray interactions through photoelectric absorption with Bi_2_O_3_ [56]. As a result, the 150 kV X-rays, which had an average energy of 118 keV, could better interact with Bi_2_O_3_ than the 100 kV (83.3 keV) X-rays, resulting in noticeably higher shielding properties of the Bi_2_O_3_/NR composites for the 150 kV X-rays.

### 3.5. Mechanical Properties

**Figure 7:**
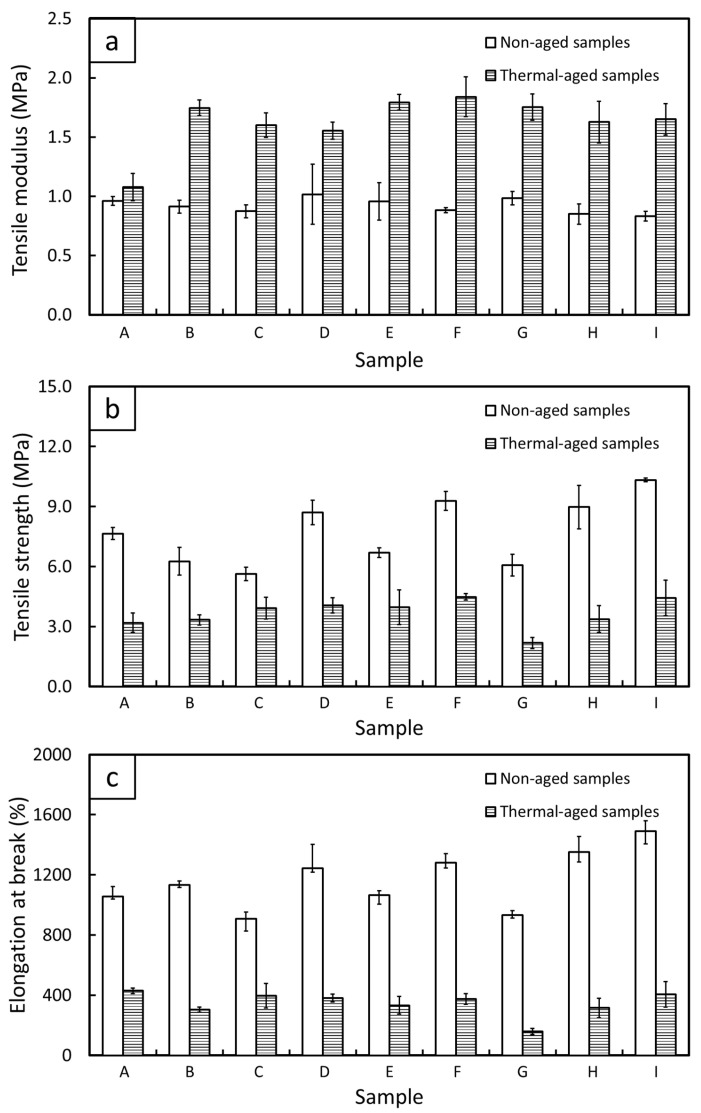
Mechanical properties of single-layered (sample-A) and multi-layered (sample-B–sample-I) Bi_2_O_3_/NR composites, both with and without thermal aging: (**a**) tensile modulus at 100% elongation, (**b**) tensile strength, and (**c**) elongation at break. The error bars represent standard deviations of the mean.

Mechanical properties, consisting of tensile modulus at 100% elongation (M100), tensile strength (TS), and elongation at break (EB), of single-layered (sample-A) and multi-layered (sample-B to sample-I) Bi_2_O_3_/NR composites are shown in Figure 7. The results indicated that the M100 values for all samples were relatively the same (close to 1 MPa), while the TS and EB values in sample-D, sample-F, sample-H, and sample-I were noticeably higher than those for other samples. The small variation in the M100 values for all samples could have been due to the theoretical numbers of Bi_2_O_3_ for a given volume being designed to be the same (Section 2.2), resulting in similar overall rigidity in the M100 values for all composites. On the other hand, sample-D, sample-F, sample-H, and sample-I, which had neat NR in both outer layers, had relatively higher TS and EB values compared with the other designs, reaching as high as 10.33 MPa and 1490% in TS and EB, respectively. These enhanced mechanical properties in the above samples were observed because the neat NR layers in the outer layers efficiently transferred external forces from the inner layers that contained Bi_2_O_3_, resulting in enhanced abilities of the composites to withstand as well as to elongate along the direction of the applied forces. The enhancement of the mechanical properties through the multi-layered structures found in this work agreed with other reports that indicated substantial improvement in the tensile properties of multi-polymer sandwich structures [57].

The effects of thermal aging on the mechanical properties of single-layered (sample-A) and multi-layered (sample-B to sample-I) Bi_2_O_3_/NR composites are also shown in Figure 7, which indicate that thermal aging led to an increase in M100 but decreases in TS and EB for all samples. The increases in M100 were due to the continuing curing process in the NR composites due to the additional heat that further crosslinked NR molecular chains with sulfur, hence increasing the overall rigidity and M100 values of the composites. Notably, the increases in M100 in sample-B to sample-I were more pronounced than that for sample-A since the percentages of torque/crosslink density in the Bi_2_O_3_/NR layers of the former samples were roughly 56–59% of their respective maximum values prior to thermal aging (Figure 2), resulting in greater opportunity for the torque/crosslink density to be enhanced in the former [52]. On the other hand, the TS and EB values of the thermal-aged samples were substantially reduced in comparison to non-aged samples, which could have been due to the initiation of chain scissions (caused by thermal oxidation) that shortened the NR molecular chains and degraded the NR composites, lowering the abilities of the composites to transfer and to withstand the supplied force, thus decreasing the TS and EB properties of all samples [58,59].

### 3.6. Correlations of Mechanical and X-Ray Shielding Properties

**Figure 8:**
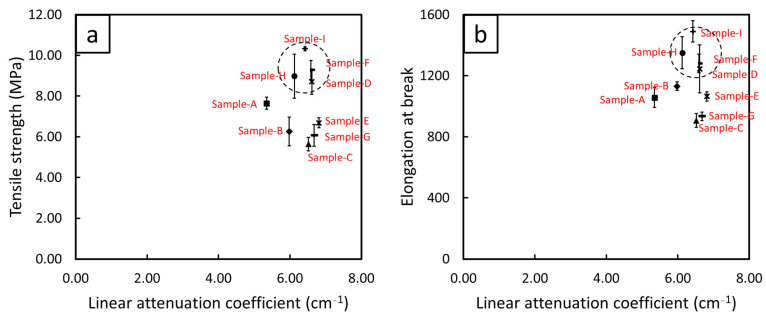
Correlations for single-layered (sample-A) and multi-layered (sample-B to sample-I) Bi_2_O_3_/NR composites between linear attenuation coefficient and: (**a**) tensile strength, and (**b**) elongation at break. The dotted circles enclose samples with relatively higher combined mechanical and X-ray shielding properties.

Figure 8 shows correlations between the mechanical and X-ray shielding properties for single-layered (sample-A) and multi-layered (sample-B to sample-I) Bi_2_O_3_/NR composites. The results revealed that after simultaneously determining values of TS/EB and µ for all samples, sample-D, sample-F, sample-H, and sample-I (enclosed inside circles in Figure 8) had relatively higher combined properties than those for the other samples. As shown and explained in Section 3.4, all the multi-layered samples had higher X-ray shielding properties than the single-layered one due to the lower build-up factor (B) values in the formers that resulted in less transmitted X-rays and thus larger measured µ values [60,61]. In addition, Section 3.5 revealed that samples with neat NR layers on both the outer sides (sample-D, sample-F, sample-H, and sample-I) had greater ability to transfer external forces from the inner layers that contained Bi_2_O_3_, leading to higher overall values of the mechanical properties. As a result, based on the combined properties, it could be concluded that the multi-layered structures (sample-D, sample-F, sample-H, and sample-I) were suitable designs to enhance both the mechanical and X-ray shielding properties of Bi_2_O_3_/NR composites compared with the common, single-layered structure.

## 4. Conclusions

Relevant properties, consisting of tensile strength, X-ray attenuation, %swelling, density, and morphology, of single-layered (sample-A) and multi-layered (sample-B–sample-I) Bi_2_O_3_/NR composites were investigated in this work to determine potential enhancements in the above properties through multi-layered structures, which could prolong the lifetime and widen the possible applications of the composites. The thorough investigation revealed that that the multi-layered structures had higher X-ray shielding properties than the single-layered one, while the samples with neat NR layers on both outer sides (sample-D, sample-F, sample-H, and sample-I) had higher tensile strength and elongation at break than the other samples. Hence, it could be concluded, based on the combined results of mechanical and X-ray shielding properties, that the properties of interest could be enhanced through multi-layered structures, with more pronounced effects in the samples having neat NR as their outer layers. In addition, this work investigated the effects of thermal aging for all samples. The aging results indicated that thermal aging led to an increase in the tensile modulus at 100% elongation but a decrease in the percentage of swelling, tensile strength, and elongation at break, with respect to non-aged samples. In conclusion, the outcomes from this work not only presented methods to enhance the mechanical and X-ray shielding properties of Bi_2_O_3_/NR composites through multi-layered structures but also provided knowledge and optimum processes to prepare multi-layered products for other applications.

## Figures and Tables

**Table 1:** Material formulation (chemical name, content, and role) of multi-layered Bi_2_O_3_/NR composites [40].

Chemical Name	Content (phr)	Role
Natural rubber (STR 20)	100 parts by weight	Main matrix
Zinc Oxide (ZnO) Stearic Acid	5 2	Activator Activator
Mercaptobenzothiazole (MBT)	2	Accelerator
Diphenylguanidine (DPG)	1	Accelerator
Paraffinic wax	40	Plasticizer
Bismuth oxide (Bi_2_O_3_)	0, 200, 333, 385, 504, 727, 1034 *	Radiation protective filler

* Contents of Bi_2_O_3_ were determined using the procedure outlined in Section 2.2.

**Table 2:** Sample codes with details of number of layers, thickness of each layer, and Bi_2_O_3_ content in each layer.

Sample	Number of Layers	Thickness of Each Layer (mm)	Bi_2_O_3_ Content in Layer (phr)				
			1	2	3	4	5
A	1	10.0	200	-	-	-	-
B	2	5.0	0	504	-	-	-
C	3	3.3	333	0	333	-	-
D	3	3.3	0	1034	0	-	-
E	4	2.5	0	504	0	504	-
F	4	2.5	0	504	504	0	-
G	4	2.5	504	0	0	504	-
H	5	2.0	385	0	385	0	385
I	5	2.0	0	727	0	727	0

**Table 3:** Torque characteristics, consisting of minimum torque (M_L_), maximum torque (M_H_), and torque difference (M_H_ − M_L_), of neat NR and Bi_2_O_3_/NR composites with varying Bi_2_O_3_ contents from 200 to 1034 phr.

Bi_2_O_3_ Content (phr)	Torque (N·m)		
	M_L_	M_H_	M_H_ − M_L_
0	0.07	0.73	0.66
200	0.11	0.96	0.85
333	0.14	1.32	1.18
385	0.12	1.33	1.21
504	0.13	1.58	1.45
727	0.20	1.97	1.77
1034	0.25	2.85	2.60

**Table 4:** Scorch times (t_s2_) and cure times (t_c10_, t_c50_, t_c90_, and t_c95_) for Bi_2_O_3_/NR composites with varying Bi_2_O_3_ contents from 0 to 1034 phr.

Bi_2_O_3_ Content (phr)	Scorch Time and Cure Time (min)				
	t_s2_	t_c10_	t_c50_	t_c90_	t_c95_
0	0.66	0.42	1.01	2.94	4.28
200	0.72	0.52	1.23	4.79	9.93
333	0.60	0.48	1.40	8.92	15.22
385	0.60	0.48	1.46	8.83	15.30
504	0.53	0.45	1.50	9.25	15.98
727	0.48	0.45	1.57	9.62	15.90
1034	0.38	0.43	1.58	10.73	19.56

**Table 5:** Densities (±standard deviation) of single-layered and multi-layered Bi_2_O_3_/NR composites.

Sample	Density (g/cm^3^)
A	1.96 ± 0.01
B	1.98 ± 0.01
C	2.00 ± 0.01
D	2.03 ± 0.01
E	2.00 ± 0.01
F	1.97 ± 0.01
G	2.04 ± 0.01
H	1.97 ± 0.01
I	1.96 ± 0.01

**Table 6:** Roughness of fractured surfaces for single-layered (sample-A) and multi-layered (sample-B to sample-I) Bi_2_O_3_/NR composites. The results are shown as the mean ± standard deviation of the mean.

Sample	Layer	Roughness (µm)
A	200-phr Bi_2_O_3_/NR	19.2 ± 6.1
B	Neat NR	3.7 ± 1.2
	504-phr Bi_2_O_3_/NR	17.7 ± 3.0
C	Neat NR	17.5 ± 6.2
	333-phr Bi_2_O_3_/NR	29.2 ± 5.2
D	Neat NR	1.2 ± 0.2
	1034-phr Bi_2_O_3_/NR	12.5 ± 0.7
E	Neat NR	2.1 ± 1.1
	504-phr Bi_2_O_3_/NR	26.2 ± 5.8
F	Neat NR	5.4 ± 1.7
	504-phr Bi_2_O_3_/NR	10.5 ± 3.6
G	Neat NR	12.2 ± 1.6
	504-phr Bi_2_O_3_/NR	20.0 ± 0.5
H	Neat NR	12.4 ± 2.6
	385-phr Bi_2_O_3_/NR	20.2 ± 2.1
I	Neat NR	2.0 ± 0.5
	727-phr Bi_2_O_3_/NR	4.6 ± 1.5

## Data Availability

The data presented in this study are available on request from the corresponding author.
